# Fluid management and vasopressor use during colorectal surgery: the search for the optimal balance

**DOI:** 10.1007/s00464-023-09980-1

**Published:** 2023-05-01

**Authors:** Daitlin E. Huisman, Boukje T. Bootsma, Erik W. Ingwersen, Muriël Reudink, Gerrit D. Slooter, Jurre Stens, Freek Daams, the LekCheck Study group, Rudi M. H. M. H. Roumen, Stefanus J. van Rooijen, Wim Bleeker, Laurents P. S. Stassen, Audrey Jongen, Carlo V. Feo, Simone Targa, Niels Komen, Hidde M. Kroon, Tarik Sammour, Emmanuel A. G. L. Lagae, Aalbert K. Talsma, Johannes A. Wegdam, Tammo S. de Vries Reilingh, Bob van Wely, Marie J. van Hoogstraten, Dirk J. A. Sonneveld, Emiel G. G. Verdaasdonk

**Affiliations:** 1 Department of Surgery, Amsterdam University Medical Centers, Location VUmc, Amsterdam, The Netherlands; 2 Department of Surgery, Máxima Medical Center, Veldhoven/Eindhoven, The Netherlands; 3 Department of Anesthesiology, Amsterdam University Medical Centers, Location VUmc, Amsterdam, The Netherlands

**Keywords:** Colorectal anastomotic leakage, Vasopressors, Fluid management

## Abstract

**Background:**

Although it is known that excessive intraoperative fluid and vasopressor agents are detrimental for anastomotic healing, optimal anesthesiology protocols for colorectal surgery are currently lacking.

**Objective:**

To scrutinize the current hemodynamic practice and vasopressor use and their relation to colorectal anastomotic leakage.

**Design:**

A secondary analysis of a previously published prospective observational study: the LekCheck study.

**Study setting:**

Adult patients undergoing a colorectal resection with the creation of a primary anastomosis.

**Outcome measures:**

Colorectal anastomotic leakage (CAL) within 30 days postoperatively, hospital length of stay and 30-day mortality.

**Results:**

Of the 1548 patients, 579 (37%) received vasopressor agents during surgery. Of these, 201 were treated with solely noradrenaline, 349 were treated with phenylephrine, and 29 received ephedrine. CAL rate significantly differed between the patients receiving vasopressor agents during surgery compared to patients without (11.8% vs 6.3%, *p* < 0.001). CAL was significantly higher in the group receiving phenylephrine compared to noradrenaline (14.3% vs 6%, *p* < 0.001). Vasopressor agents were used more often in patients treated with Goal Directed Therapy (47% vs 34.6%, *p* < 0.001). There was a higher mortality rate in patients with vasopressors compared to the group without (2.8% vs 0.4%, *p* = 0.01, OR 3.8). Mortality was higher in the noradrenaline group compared to the phenylephrine and those without vasopressors (5% vs. 0.4% and 1.7%, respectively, *p* < 0.001). In multivariable analysis, patients with intraoperative vasopressor agents had an increased risk to develop CAL (OR 2.1, CI 1.3–3.2, *p* = 0.001).

**Conclusion:**

The present study contributes to the evidence that intraoperative use of vasopressor agents is associated with a higher rate of CAL. This study helps to create awareness on the (necessity to) use of vasopressor agents in colorectal surgery patients in striving for successful anastomotic wound healing. Future research will be required to balance vasopressor agent dosage in view of colorectal anastomotic leakage.

Although research has shown that both excessive and restrictive intraoperative fluid administration as well as vasopressor use could possibly be detrimental for colonic anastomotic healing (CAL) [1–4], optimal anesthesiology protocols for colorectal surgery are currently lacking. Multiple strategies have been reported: restrictive fluid therapy, also called near-zero fluid balance, consists of replacing only the fluid that is lost during surgery, while Goal Directed Therapy (GDT) refers to the hemodynamic optimization during surgery by titrating fluids, using vasopressors and/or inotropes to reach predefined hemodynamic goals to maintain sufficient perfusion [5]. The literature is equivocal on the superiority of either of the two regimens. Earlier studies have suggested that restrictive fluid therapy is associated with a shorter length of stay and better short-term outcomes compared to a liberal fluid regimen [4, 6]. Few studies have investigated GDT in colorectal surgery with CAL as endpoint with a meta-analysis not showing a benefit [7]. For vasopressor use, the fourth updated Enhanced Recovery After Surgery (ERAS) Society guideline recommends intraoperative vasopressor use, although based on studies that were not colorectal surgery specific [8–10]. Therefore, this study aimed to investigate the current practice of intraoperative hemodynamic care in colorectal patients, including the current fluid management and vasopressor use and their relation to CAL.

## Methods

### Study design and population

From January 2016 to December 2019, 14 hospitals (11 in the Netherlands, one in Belgium, one in Italy and one in Australia) participated in the LekCheck study, a multicenter prospective cohort study [11]. In the LekCheck study, consecutive adult patients undergoing colorectal surgery with primary anastomosis were enrolled. A detailed description of the study design and the main results have been published previously. Informed consent was obtained from all patients. The administration of vasopressor agents was registered (yes/no) during the creation of the anastomosis intraoperatively. If at that moment the patient was receiving vasopressors, the answer yes was given and the type of drugs and dosage was noted. For the current analysis, patients treated with ephedrine were excluded from the analysis. In the noradrenaline patient group, the patients were solely treated with noradrenaline during surgery.

### Data collection

The following variables were collected preoperatively: age, sex, diabetes mellitus (yes/no), body mass index (BMI), steroid use, intoxications (smoking status and alcohol), American Society of Anesthesiologist (ASA) score, benign or malignant disease as the indication for surgery, tumor node and metastasis (TNM) and American Joint Committee on Cancer (AJCC) stage, neoadjuvant therapy, tumor distance from the anal verge (AV) and preoperative hemoglobin level (HB). Intraoperatively the following parameters were collected: blood glucose level, use of epidural anesthesia, use and type and dosage of vasopressors, volume of blood loss, fluid administration, body temperature in degrees Celsius, mean arterial pressure (MAP), oxygen saturation, the occurrence of an intraoperative event (e.g., hypoxic events, hypertension, hypercarbia, bradycardia, hypotension, embolism, reanimation, formation of a stoma and stoma type, more extensive resection than planned, serosa lesions, bladder and ureteral injuries, intraoperative bleeding, splenectomy) and the assessment of fecal contamination. Postoperatively, the following variables were collected: the occurrence of CAL within 30 days, length of hospital stay and mortality.

### Outcomes

Primary outcome was CAL. CAL was defined according to Reisinger, et al.: “Clinically relevant anastomotic leakage is defined as extra luminal presence of contrast fluid on contrast-enhanced CT scans and/or leakage when relaparotomy was performed, requiring re-intervention or treatment.” [12, 13]. Secondary outcomes were demise of the patient during hospital stay and length of hospital stay.

### Missing data

Missing data were imputed using predictive mean matching with 10 iterations. Variables with more than 30% missing data were excluded.

### Cutoff values and statistics

Patient characteristics and intraoperative variables between patients with and without vasopressors were compared. Subsequently, within the vasopressor group, patients who received noradrenaline were compared to patients with phenylephrine. Dichotomous and categorical data were expressed in percentages and frequencies. Continuous variables were presented as means or medians. Dichotomous and categorical data were expressed in percentages and frequencies. Continuous intraoperative parameters were prior to inclusion dichotomized to create a composite score. The preferable cutoff values were derived from a previously published review [6]. Only exceptions were a low hemoglobin value below 7 mmol/L, hyperglycemia defined as a glucose level of > 10 mmol/L and blood loss defined as > 500 mL during the procedure, making these cutoff values as unnegotiable risk factors. Dichotomous data were compared using the *X*^*2*^ test. Continuous data were compared using the Student’s *t* test or the Mann–Whitney *U* test (skewed distribution). The primary and secondary outcomes were analyzed in a univariate analysis with the administration of vasopressor agents as the independent and the outcome as the dependent variable. Then, a multivariable analysis was performed to adjust for confounders. Relevant confounders were defined as preoperative or intraoperative variables which altered the B value of the logistic regression model with more than 10%. A *p* value < 0.05 was regarded as statistically significant. Results are reported as odds ratios (OR) and 95% confidence intervals (CIs). Data were analyzed with Statistical Package for the Social Sciences software (SPSS, version 26).

## Results

Data from 1821 colorectal patients were collected, 180 non-elective patients were excluded and another 93 due were excluded to incomplete data. A total of 1548 patients were found eligible. The median age was 69 years (range 21–95 years) and 803 (52%) of the patients were male. Of the 1548 patients, 579 (37%) received vasopressor agents during surgery. From the 579 patients in the vasopressor agents group, 201 were treated with solely noradrenaline, 349 were treated with phenylephrine, and 29 received ephedrine. There were 129 (8.3%) CAL cases.

### Patient characteristics and intraoperative factors

Patients with vasopressors during surgery were more often older than 70 years (56% vs. 45%, *p* < 0.001), had more frequently diabetes mellitus (19% vs. 13%, *p* = 0.001), had more frequently a colorectal malignant diagnosis (86% vs. 80%, *p* = 0.003) and less often a tumor within 15 cm from the AV (27% vs. 36%, *p* = 0.016). Significant intraoperative factors differing between patients with and without vasopressor agents were epidural analgesia (37% vs. 31%, *p* = 0.010), GDT (26% vs. 16%, *p* < 0.001), blood loss > 500 mL (7% vs. 2%, *p* = 0.003), blood transfusion (4.5% vs. 1.1%, *p* < 0.001), low hemoglobin (21% vs 12%, *p* < 0.001) and fluid administration < 500 mL/h (41% vs. 48%, *p* = 0.036). See Tables 1 and 2.

**Table 1:** Baseline characteristics of vasopressor agents (*n* = 1548)

Variable	Vasopressor agents (*n* = 579)		No vasopressor agents (*n* = 969)		*p* value
		Missing		Missing	
Sex (male)	290 (50%)		513 (53%)		0.150
Age (years) *	72 (24–92)		68 (21–95)		** < 0.001**
≥ 70	319 (56%)		438 (45%)		
Body mass index ≥ 30 kg/m^2^	128 (23%)		190 (20%)		0.113
ASA classification					** < 0.001**
≥ 3	197 (35%)		187 (19%)		
Diabetes mellitus	107 (19%)	*n* = 7	123 (13%)	*n* = 5	**0.001**
Intoxications					
Current smoker	62 (12%)	*n* = 19	124 (13%)	*n* = 45	0.160
Pack years ≥ 15 years	117 (27%)	*n* = 23	224 (28%)	*n* = 43	0.435
Alcohol intake ≥ 3 units/day	27 (5%)	*n* = 21	45 (5%)	*n* = 35	0.511
Steroid use (excl. inhalers)	15 (2%)	*n* = 9	25 (3%)	*n* = 5	0.542
Disease				*n* = 6	**0.003**
Malignant	491 (86%)		774 (80%)		
Benign	83 (14%)		194 (20%)		
Neoadjuvant therapy		*n* = 20		*n* = 47	0.057
None	494 (89%)		795 (86%)		
5 × 5 radiotherapy	30 (6%)		72 (8%)		
Chemotherapy	14 (3%)		19 (2%)		
Chemoradiotherapy	11 (2%)		36 (4%)		
Distance of tumor from AV < 15 cm	88 (27%)		213 (36%)		**0.004**
Pathological TNM stage		*n* = 7		*n* = 7	0.458
I (T1-2N0M0)	217 (37%)		339 (35%)		
II (T3-4N0M0)	119 (21%)		169 (18%)		
III (T1-4N1-2M0)	127 (22%)		203 (21%)		
IV (T1-4N1-2M1)	21 (4%)		56 (6%)		

Bold values indicate significance of *p* value (*p* < 0.005)

Data are presented as number (%) unless stated otherwise

*ASA* American Society of Anesthesia score, *TNM* Tumor, node and metastasis classification, *AV* Anal verge

A *p* < 0.05 was considered statistically significant

*Data are presented as medians (range)

**Table 2:** Surgery-related factors and risk for vasopressor agents

Variable	Vasopressor agents (*n* = 579)		No vasopressor agents (*n* = 969)		*p* value
		Missing		Missing	
Could vasopressor agents be stopped?	322 (56%)				
Epidural	209 (37%)		291 (31%)		**0.010**
Goal Directed Therapy	145 (26%)		160 (16%)		** < 0.001**
Intraoperative event*	92 (16%)		126 (13%)		0.065
Temperature 36 degrees	109 (19%)	*n* = 1	195 (20%)	*n* = 7	0.272
Glucose > 10 mmol/L	52 (9%)	*n* = 13	76 (8%)	*n* = 49	0.299
Antibiotics < 15 or > 60 min prior incision	132 (24%)	*n* = 17	221 (24%)	*n* = 38	0.482
Blood loss > 500 mL	31 (7%)	*n* = 35	37 (2%)	*n* = 42	**0.003**
Oxygen < 94%	13 (22%)		14 (15%)		0.173
Mean arterial pressure < 65	53 (9%)		86 (9%)		0.463
Urine production < 30 mL/h	98 (18%)		156 (17%)		0.350
OR time > 180 min	167 (29%)		288 (30%)		0.379
Hemoglobin					**0.001**
Men < 7 mmol/L	48 (9%)		42 (4%)		< 0.001
Woman < 6.5 mmol/L	111 (21%)		106 (12%)		
Surgical approach					0.113
Open	95 (16%)		136 (14%)		
Laparoscopy	451 (78%)		779 (80%)		
Laparoscopy with conversion	31 (5%)		53 (5%)		0.223
Fluid administration					0.169
< 250 mL/h	89 (15%)		120 (12%)		
250–1000 mL/h	453 (78%)		773 (80)		
> 1000 mL/h	37 (6%)		76 (8%)		
Fluid administration > 500 mL/h	236 (41%)		465 (48%)		**0.003**
Blood transfusion					** < 0.001**
1 packed cells	21 (4%)		10 (1%)		
2 packed cells	3 (0.5%)		1 (0.1%)		

Bold values indicate significance of *p* value (*p* < 0.005)

Data are presented as number (%) or as medians (range) for categorical and continuous variables, respectively. N is number of inclusions if due to missing data this deviates from total

A *p* < 0.05 was considered statistically significant

*Intraoperative events include: hypoxic events, hypertension, hypercarbia, bradycardia, hypotension, embolism, reanimation, more extensive resection than planned, serosa lesions, bladder and ureteral injuries, intraoperative bleeding, splenectomy or bleeding

Table 3 shows the different characteristics between patients with intraoperative administration of noradrenaline or phenylephrine. Patients with noradrenaline more often had diabetes mellitus (24% vs. 15%, *p* = 0.006) and more often a tumor distance to the AV of < 15 cm (12% vs. 8%, *p* = 0.027). In univariate analysis, the intraoperative factors associated with noradrenaline patients were: use of an epidural (51% vs. 26%, *p* < 0.001), GDT (33% vs. 21%, *p* = 0.002), intraoperative event (21% vs. 13%, *p* = 0.027), temperature < 36.0 degrees Celsius (26% vs. 15%, *p* = 0.002), blood loss > 500 mL (10% vs. 5%, *p* = 0.037), mean arterial pressure (MAP) < 65 mmHg (12% vs. 7%, *p* = 0.029), operation time > 180 min (36% vs 26%, *p* = 0.010), conversion from laparoscopic to open surgery (9% vs. 4%, *p* < 0.001), fluid administration > 500 mL/h (48% vs. 67%, *p* < 0.001), high blood glucose > 10 mmol/L (16% vs. 6%, *p* < 0.001) and a blood transfusion given (7.5% vs. 2.6%, *p* = 0.048), see Table 4. GDT was followed in 19% (*n* = 305) of all patients. Vasopressors were used significantly more in these patients (47% in patients with GDT vs. 34.6% without, *p* < 0.001), see Table 5.

**Table 3:** Baseline characteristics of noradrenaline or phenylephrine (*n* = 550)

Variable	Noradrenaline (*n* = 201)		Phenylephrine (*n* = 349)		*p* value
		Missing		Missing	
Sex (male)	97 (48%)		182 (52%)		0.215
Age (years)*	71 (31–95)		72 (24–94)		0.455
≥ 70	115 (58%)		193 (57%)		
Body mass index ≥ 30 kg/m^2^	39 (20%)		82 (24%)		0.153
ASA classification					0.519
≥ 3	70 (35%)		121 (35%)		
Diabetes mellitus	48 (24%)		51 (15%)	n = 6	**0.006**
Intoxications					
Current smoker	23 (12%)	*n* = 10	34 (11%)	*n* = 23	0.348
Pack years ≥ 15 years	41 (32%)	*n* = 12	70 (25%)	*n* = 27	0.060
Alcohol intake ≥ 3 units/day	12 (6%)	*n* = 9	14 (4%)	*n* = 17	0.219
Steroid use (excl. inhalers)	6 (3%)		9 (3%)		0.504
Disease				*n* = 5	0.423
Malignant	171 (85%)		296 (86%)		
Benign	30 (15%)		48 (14%)		
Neoadjuvant therapy		*n* = 6		*n* = 19	0.051
None	167 (86%)		305 (93%)		
5 × 5 radiotherapy	16 (8%)		11 (3%)		
Chemotherapy	6 (3%)		8 (2%)		
Chemoradiotherapy	5 (3%)		4 (1%)		
Distance of tumor from AV < 15 cm	25 (12%)		28 (8%)		**0.027**
Pathological TNM stage		*n* = 7		*n* = 7	0.129
I (T1-2N0M0)	62 (31%)		141 (40%)		
II (T3-4N0M0)	58 (29%)		60 (17%)		
III (T1-4N1-2M0)	41 (20%)		79 (23%)		
IV (T1-4N1-2M1)	6 (3%)		13 (4%)		

Bold values indicate significance of *p* value (*p* < 0.005)

Data are presented as number (%) unless stated otherwise

*ASA* American Society of Anesthesia score, *TNM* Tumor, node and metastasis classification, *AV* Anal verge

A *p* < 0.05 was considered statistically significant

*Data are presented as medians (range)

**Table 4:** Surgery-related factors and use noradrenaline or phenylephrine (*n* = 550)

Variable	Noradrenaline (*n* = 201)		Phenylephrine (*n* = 349)		*p* value
		Missing		Missing	
Could vasopressor agents be stopped?	68 (34%)		238 (68%)		** < 0.001**
Epidural	102 (51%)		91 (26%)		** < 0.001**
Goal directed therapy	63 (33%)		74 (21%)		**0.002**
Intraoperative event*	41 (21%)		48 (13%)		**0.027**
Temperature < 36 degrees	51 (26%)	*n* = 1	53 (15%)		**0.002**
Glucose > 10 mmol/L	30 (16%)	*n* = 11	21 (6%)	*n* = 1	< 0.001
Antibiotics < 15 or > 60 min prior incision	52 (27%)	*n* = 10	71 (21%)	*n* = 7	0.057
Blood loss > 500 mL	18 (10%)	*n* = 11	15 (5%)	*n* = 28	**0.037**
Oxygen < 94%	5 (3%)		8 (2%)		0.544
Mean arterial pressure < 65 mmhg	24 (12%)		24 (7%)		**0.029**
Urine production < 30 mL/h	19 (9%)		78 (22%)		** < 0.001**
OR time > 180 min	73 (36%)		92 (26%)		**0.010**
Hemoglobin					0.284
Men < 6.5, woman < 6 mmol/L	16 (8%)		31 (10%)		
< 7 mmol/L	39 (19%)		67 (21%)		**0.340**
Surgical approach					** < 0.001**
Open	55 (16%)		32(14%)		
Laparoscopy	127 (63%)		303 (87%)		
Laparoscopy with conversion	18 (9%)		13 (4%)		0.223
Fluid administration					0.103
< 250 mL/h	26 (13%)		62 (18%)		
250–1000 mL/h	158 (79%)		270 (77%)		
> 1000 mL/h	17 (8%)		17 (5%)		
Fluid administration > 500 mL/h	97 (48%)		232 (67%)		** < 0.001**
Blood transfusion					**0.048**
1 packed cells	13 (7%)		8 (2%)		
2 packed cells	1 (0.5%)		2 (0.6%)		

Bold values indicate significance of *p* value (*p* < 0.005)

Data are presented as number (%) or as medians (range) for categorical and continuous variables, respectively. N is number of inclusions if due to missing data this deviates from total

*Intraoperative events include: hypoxic events, hypertension, hypercarbia, bradycardia, hypotension, embolism, reanimation, more extensive resection than planned, serosa lesions, bladder and ureteral injuries, intraoperative bleeding, splenectomy or bleeding. A *p* < 0.05 was considered statistically significant

**Table 5:** Univariate analysis of Goal Directed Therapy

	GDT (*n* = 305)	No GDT (*n* = 1221)	*p* value
Vasopressor	145 (47.5%)	422 (34.6%)	< 0.001
Urine output 30 mL/h	53 (18%)	201 (18%)	0.530
Blood loss > 500 mL	16 (5.9%)	51 (4.6%)	0.238
Noradrenaline	63 (20.6%)	127 (10.4%)	
Phenylephrine	74 (24.3%)	275 (22.5%)	
Fluid < 250 mL/h	46	161	
Fluid > 250– < 500 mL/h	234	977	
Fluid > 1000 mL/h	25	83	

In the vasopressor agents group, there were 68 CAL versus 61 CAL in the group without vasopressors (11.7% vs 6.3%, *p* < 0.001). In the phenylephrine group, there were 50 CAL and in the noradrenaline group 18 CAL (14.3% vs 6%, *p* = 0.002). Multivariable analysis showed that intraoperative vasopressor agents had an OR of 2.1 to develop CAL (CI 1.3–3.2), see Table 6. Multivariable analysis also showed that phenylephrine had a higher risk of developing CAL compared to noradrenaline (OR 4.2; CI 1.9–8.6), see Table 7. There were no significant differences between fluid administration and vasopressive agents in colon (*p* = 0.73) or rectal surgery (*p* = 0.45) in multivariate analysis. Only in malignant resections, multivariable analysis showed that the odds of receiving vasopressors during surgery is twice as high versus benign surgery (OR 2.0, CI 1.2–3.3, *p* = 0.004). Open surgery is not independently associated with a higher risk of CAL in multivariate analysis. However, a multivariate analysis found that open versus laparoscopic surgery reduced the chance of receiving less than 500 mL/h of fluids (OR 0.5, CI 0.3–0.8, *p* = 0.010).

**Table 6:** Multivariate analysis of postoperative outcomes with vasopressor agents and non-vasopressors agents

	Vasopressor (579)	No Vasopressor (971)	Total (*n* = 1549)	Univariate analysis		Multivariate analysis	
				OR (95% CI)	*P* value	OR (95% CI)	*p* value
Anastomotic leakage	68 (11.8%)	61 (6.3%)	129 (8.3%)	2.0 (1.3–2.8)	< 0.001	2.1 (1.3–3.2)*	0.001*
Mortality	16 (2.8%)	4 (0.4%)	129 (8.3%)	6.9 (2.3–21)	0.001	5.1 (1.6–16)**	0.005**
Length of stay	M = 5	M = 4	M = 4	1.5 (1.2–1.9)	0.001	1.2 (0.9–1.5)***	0.187

*OR* Odds ratio, *CI* Confidence interval, *M* Median

*In the multivariate analyses adjusted for epidural, pack years > 15 and low hemoglobin

**In the multivariate analyses adjusted for pack years > 15 and low hemoglobin

***In the multivariate analyses adjusted for ASA > 3 and low hemoglobin

**Table 7:** Multivariate analysis of postoperative outcomes with noradrenaline and phenylephrine group

	Noradrenaline (*n* = 201)	Phenylephrine (*n* = 349)	Total (*n* = 1549)	Univariate analysis		Multivariate analysis	
				OR (95% CI)	*p* value	OR (95% CI)	*p* value
Anastomotic leakage	68 (11.8%)	61 (6.3%)	129 (8.3%)	2.6 (1.4–5.1)	0.004	4.2 (1.9–8.6)*	< 0.001*
Mortality	10 (5%)	6 (1.7%)	129 (8.3%)	0.3 (0.1–0.9)	0.037	0.3 (0.1–0.8)**	0.019**
Length of stay	M = 6	M = 4	M = 4	0.6 (0.4–0.9)	0.024	0.6 (0.3–1.1)***	0.052***

*OR* Odds ratio, *CI* Confidence interval, *M* Median

*In the multivariate analyses adjusted for temperature < 36 degrees, low hemoglobin and OR time > 180 min

**In the multivariate analyses adjusted for ASA > 3

***In the multivariate analyses adjusted for low hemoglobin, pack years > 15, intraoperative event and epidural

There was a 1.3% mortality rate in the total study population. Sixteen of these 20 patients received vasopressor agents during surgery (2.8% vs 0.4%, *p* < 0.001). Within the vasopressor agents group, 30-day mortality rate was 5% in the noradrenaline group compared to 1.7% in the phenylephrine group (*p* = 0.002). Multivariable analysis showed that the use of vasopressors had an OR of 5.1 for mortality compared to patients without intraoperative vasopressor agent use (CI 1.6–16), see Table 6.

The median length of hospital stay was not different for patients with or without vasopressors. There was no significant association in the occurrence of CAL between patients treated with or without GDT.

## Discussion

This study scrutinized the current practice of intraoperative hemodynamic care in colorectal patients. The prospectively collected data showed that the use of intraoperative vasopressor agents was related to a significantly higher CAL and mortality rate.

This could be due to the physiology of vasopressors during a procedure not being beneficial for the healing of the anastomosis. The factors that showed a relation to vasopressor agent use are: age > 70 years, diabetes mellitus, malignant diseases, distance of tumor to AV < 15 cm, epidural use, GDT, blood loss > 500 mL, blood transfusion, low hemoglobin and fluid administration < 500 mL/h. The question is whether the vasopressor agents themselves exert a detrimental effect on CAL and survival, or that this effect should be contributed to the circumstances that led to the decision to administer vasopressor agents.

The results of this study are compliant with those of the study by Adanir et al. [2], who showed that vasopressors appeared to increase the risk of CAL due to vasoconstriction, deterioration of microcirculation and possibly local hypoxia. Similarly, Choudhuri et al. [3] found that patients who required inotropic support during surgery had a four times higher risk of CAL.

In the RELIEF study [14], the largest trial to date of perioperative fluid management, restrictive fluid management was associated with a higher rate of acute kidney injury. Myles et al. showed that restrictive fluid regimen was not associated with a higher rate of disability-free survival versus liberal fluid management [15]. Over the past decade, patients undergoing colorectal surgery were increasingly subject to a restrictive fluid management [16–18]. Studies showed that a liberal fluid regime could lead to an increase in interstitial volume, cardiopulmonary dysfunction, inflammation, edema and impaired tissue oxygenation. This is due to hypervolemia causing shedding of the endothelial glycocalyx and therefore affecting the vascular permeability [19]. An optimal threshold for stroke volume variation to prevent this still needs to be determined [20]. Anastomotic wound healing is adversely affected by intestinal edema [17]. Consequently, The American Society for Enhanced Recovery and Perioperative Quality Initiative created a framework for perioperative fluid management and concluded that GDT is safe in the majority of colorectal patients [18, 21]. Correct patient allocation for GDT, however, is still under debate. GDT could be the solution for not giving too much fluid during surgery, but the benefits in outcome have yet to be proven [21, 22]. The current study found that in patients treated with GDT more vasopressors were used. It could be that the avoidance of excessive fluid turned into an inordinate restrictive regime [23]. An other possibility is that GDT is more often applied in higher risk patients. Recent studies show that a restrictive fluid management and GDT also have their drawbacks [21, 22], possibly because more vasopressors are used to compensate for blood pressure and heart rate.

There are some limitations to this study. Despite our multivariate analysis, in which was tried to correct for patient characteristics and intraoperative variables, a firm conclusion cannot be drawn due to an unclear cause–effect relation. For instance, pre-existing risk factors, such as comorbidities and higher ASA-scores, could in itself be a cause for more vasopressor agents during surgery, while it is also known that these patients have a higher risk for developing CAL [24]. The administration of vasopressors may therefore rather be an intraoperative requirement and reflect the patient’s preoperative condition [25]. The lack of postoperative information limits the ability to draw conclusions between the outcomes and the use of vasopressors and fluid restriction postoperatively. Nevertheless, this study shows an association with CAL and mortality in patients with certain vasopressor agents. Currently, our group is mapping current anesthesia practice for colorectal surgery.

In the future, a prospective follow-up study that stratifies for intraoperative conditions could contribute to the knowledge regarding the association between specific vasopressive agents and outcomes such as CAL and mortality.

The present study contributes to the evidence that intraoperative use of vasopressor agents is associated with a higher rate of CAL. This study helps to create awareness on the (necessity to) use of vasopressor agents in colorectal surgery patients in striving for successful anastomotic wound healing. Future research will be required to balance vasopressor agent dosage in view of colorectal anastomotic leakage.

## Abbreviations

AL: Anastomotic leakage
BMI: Body mass index
ASA: American Society of Anesthesiologists
AJCC: American Joint Committee on Cancer
MAP: Mean arterial pressure
TNM: Tumor node and metastasis
ERAS: Enhanced recovery after surgery
